# Methodology for the Quality Control Process of Additive Manufacturing Products Made of Polymer Materials

**DOI:** 10.3390/ma14092202

**Published:** 2021-04-25

**Authors:** Grzegorz Budzik, Joanna Woźniak, Andrzej Paszkiewicz, Łukasz Przeszłowski, Tomasz Dziubek, Mariusz Dębski

**Affiliations:** 1 Department of Machine Design, The Faculty of Mechanical Engineering and Aeronautics, Rzeszow University of Technology, al. Powstańców Warszawy 12, 35-959 Rzeszów, Poland; gbudzik@prz.edu.pl (G.B.); lprzeszl@prz.edu.pl (Ł.P.); tdziubek@prz.edu.pl (T.D.); m.debski@prz.edu.pl (M.D.); 2 Department of Management Systems and Logistics, The Faculty of Management, Rzeszow University of Technology, al. Powstańców Warszawy 12, 35-959 Rzeszów, Poland; j.wozniak@prz.edu.pl; 3 Department of Complex Systems, The Faculty of Electrical and Computer Engineering, Rzeszow University of Technology, al. Powstańców Warszawy 12, 35-959 Rzeszów, Poland

**Keywords:** additive manufacturing, quality control management, process optimization, computing techniques, ICT systems

## Abstract

The objective of this publication is to present a quality control methodology for additive manufacturing products made of polymer materials, where the methodology varies depending on the intended use. The models presented in this paper are divided into those that are manufactured for the purpose of visual presentation and those that directly serve the needs of the manufacturing process. The authors also a propose a comprehensive control system for the additive manufacturing process to meet the needs of Industry 4.0. Depending on the intended use of the models, the quality control process is divided into three stages: data control, manufacturing control, and post-processing control. Research models were made from the following materials: RGD 720 photopolymer resin (PolyJet method), ABS M30 thermoplastic (FDM method), E-Partial photopolymer resin (DLP method), PLA thermoplastic (FFF method), and ABS thermoplastic (MEM method). The applied measuring tools had an accuracy of at least an order of magnitude higher than that of the manufacturing technologies used. The results show that the PolyJet method is the most accurate, and the MEM method is the least accurate. The findings also confirm that the selection of materials, 3D printing methods, and measurement methods should always account not only for the specificity and purpose of the model but also for economic aspects, as not all products require high accuracy and durability.

## 1. Introduction

Quality control is one of the key elements of the manufacturing process, regardless of the number of manufactured products. Depending on the stage of the technological process, methods of quality control include different activities with varying scopes, such as ensuring the correctness of designed 3D CAD models; verifying prepared process data; performing visual product control; and controlling dimensional and shape accuracy, surface geometric structure, and material internal structure, particularly in the case of safety-critical elements of vehicles and aircrafts [1,2,3,4]. Quality control processes in manufacturing systems have been the subject of numerous publications. Many of them are related to computing techniques, such as contact coordinate computing [5,6], geometric measurement and analysis using optical computing systems [7,8,9,10], and geometric accuracy analyses using volume-based methods, including computed tomography [11,12,13]. Product research that applies CT can produce comprehensive results that reveal the geometric accuracy of both the external and internal surfaces of the product, as well as its material structure. However, it is costly and time-consuming and requires specialized equipment. Therefore, CT is reserved for particular product groups, such as aircraft engine blades [14,15,16]. Quality control in the manufacturing process can be accelerated by applying gauges, but these instruments provide information relevant to a particular product dimension, for instance, the measurement necessary for the mounting or functioning of an element in a machine set [17]. One of the methods for accelerating computing is the application of automated 3D scanning systems, including hardware and software automation, based on robotized measuring sockets that apply rapid scanning procedures. A perfect solution in this case is the application of blue light 3D scanners [18], which enable the quality control of both small-dimension elements and large-dimension artifacts directly on the production line: for instance, these instruments are useful for ensuring the accuracy of vehicle bodywork.

The applied additive manufacturing models and 3D printing method affect several aspects of product quality control and should be considered when developing quality assessment processes. For the quality control of an additive manufacturing model, it is important to consider its place in the technological process. A model that has the properties of an end product will be subjected to quality control before it can be mounted or sold. Visual models are often indispensable at the stages of product conception or the determination of ergonomic characteristics [19,20], but quality control is not usually particularly strict in such cases. For the additive manufacturing of products made of polymer materials, additional mechanical or thermal-chemical processing is not usually applied. Taking this into account, after finishing 3D printing and post-processing, an element has to be subjected to thorough quality control, including both visual control and the analysis of dimensional and shape accuracy [21]. Additive manufacturing technologies that are currently applied to the elements of metal powders (SLS/SLM/DMLS/PBF) can produce semi-finished products that require further mechanical processing and, frequently, thermal-chemical processing. This results in the need for quality control of both the semi-finished product and end product of the additive manufacturing process [22]. Therefore, the material of a product, as well as the additive manufacturing method, can be an important basis for determining the appropriate quality control methodology, which is also defined in particular standards [23,24,25,26,27,28,29,30,31].

Visual prototype assessment plays a significant role in the quality control of additive manufacturing products made of polymer materials. These assessments include the analysis of model structure continuity, correctness in reference to a 3D CAD model, and the colors of elements made of numerous materials that come in various colors or external color textures [32]. Dimensional and shape accuracy must be analyzed with the appropriate tools, devices, and computing systems [18]. In the majority of cases, the dimensional accuracy of products made of polymer materials does not exceed ± 0.05 mm. For this reason, these products may be effectively evaluated with workshop measuring instruments, such as a caliper or micrometer [33]. For products with complex shapes, blue light 3D scanning systems, such as 3D scanners from the GOM Company (Braunschweig, Germany), can be applied to the computing process [34]. These systems enable the relatively rapid quality controls of products in real-time or offline with the application of, for instance, computing results saved in a data cloud. With this approach, 3D scanners can be ideal measuring instruments that simultaneously perform detailed quality control and geometric analyses, which can significantly accelerate the quality control process, particularly in the case of artifacts with complex shapes. The methodology of such an approach to quality control can be developed based on the principles of Industry 4.0, in which available data are used for multi-level and parallel control computing. The application of 3D scanning as a control tool for additive manufacturing products has yet another aspect relevant to data processing: an additive manufacturing product is frequently manufactured on the basis of a 3D CAD/3D STL model, and the STL format is primarily used in the analysis of product dimensional accuracy. In this case, data in STL format from the computing point cloud are compared with data saved in the form of a nominal STL model based on a 3D CAD model [35]. This is a significant element of the quality control methodology, a major part of which is analyzing and processing numerical data.

In the literature on this subject, the quality control of products fabricated via incremental manufacturing is most often dominated by technical aspects. However, there is still a lack of research and analysis on the management and systematic ordering of processes [12,36].

In line with the above factors, the principal objective of this publication is to present a quality control methodology for additive manufacturing products made of polymer materials, where the methodology varies depending on the intended use. In this paper, the 3D models used for quality control procedures are divided into those that are manufactured for the purpose of visual presentation and those that directly serve the needs of the manufacturing process. For each group, an algorithm of the developed quality control methodology is presented graphically. The authors also propose a comprehensive control system for the additive manufacturing process to meet the needs of Industry 4.0, which applies to both direct control by a qualified specialist and complete automation, as well as remote supervision.

The findings presented in this paper can be used in practice by academia and manufacturing companies that use additive manufacturing technologies in their production processes. In the context of Industry 4.0, the developed quality process for additive manufacturing products made from polymeric materials includes access to a large production database. In the initial phase, an image is formed during production to identify geometry deviations, and on this basis, factors that influence their formation can be determined. The measurement data obtained during inspections can be used to adjust both the geometry of the 3D CAD models and the parameters of the manufacturing processes, thus increasing the accuracy of manufactured parts. Due to its rapid response to data, an Industry 4.0 system is able to immediately react to phenomena that occur during production, which has a widespread impact on the dimensional and shape accuracy of manufactured products.

## 2. Standards Applied in 3D Printing Quality Control

With the increasing application of additive manufacturing technologies in the fabrication of end products, it became necessary to establish standards for 3D printing quality and the organization of processes within the entire delivery chain [37]. International activity in the scope of additive manufacturing technologies commenced at the end of 2011 when the Technical Committee for Additive Manufacturing, designated ISO/TC 261, was established. Since the very first days of its activity, it has worked in close cooperation and formed joint committees with ASTM F42 Additive Manufacturing Technologies. The fruits of this work are numerous standards in the ISO/ASTM series. Publications and projects developed in the field of additive manufacturing are primarily related to general principles and terminology, process categorization, profiles and research methods, computer process description (with the application of a determined standard data saving mode and data in the form of a file), and the assessment of the geometric accuracy of additive manufacturing processes.

Major publications in the field of 3D printing are as follows:

- PN/EN ISO/ASTM 52900:2017-06 Additive Manufacturing—General Principles—Terminology. This includes terms and definitions related to additive manufacturing technologies, in which physical structures (geometries) are developed by adding additional material layers.
- PN/EN ISO/ASTM 52901:2019-01 Additive Manufacturing—General Principles—Requirements relevant to parts manufactured by means of additive manufacturing (AM) processes. This document is applicable to purchasing parts produced with additive manufacturing technologies; it sets the minimum requirements that must be met for the acceptance of products. The document recommends that additional stricter requirements be determined in the course of placing an order.
- PN/EN ISO 17296-2:2016-10 Additive Manufacturing—General Principles—Part 2: Overview of process categories and feedstock. This document presents the foundations of the additive manufacturing process and a review of existing process categories. It also describes how to apply different kinds of materials to shape the geometry of a given product in different process categories.
- PN/EN ISO 17296-3:2016-10 Additive Manufacturing—General Principles—Part 3: principal characteristics and appropriate research methods. This standard is primarily dedicated to machine producers and users, feedstock and part suppliers, and recipients and customers. It contains basic requirements that ought to be applied to parts produced with additive manufacturing technologies.
- PN/EN ISO 17296-4:2016-10 Additive Manufacturing—General Principles—Part 4: Overview of data processing. This standard contains issues related to data exchange. It also describes terms and definitions applied to the exchange of information about the geometry of parts (products) manufactured with additive manufacturing.
- ISO/ASTM 52902:2019 Additive Manufacturing—Test Artifacts—Geometric capability assessment of additive manufacturing systems. This standard contains a general description of model specimen geometries, together with the quantitative and qualitative computing to be conducted on a test specimen(s) in order to assess the efficiency of additive manufacturing (AM) systems.
- ISO/ASTM TR 52912:2020 Additive manufacturing—Design—Functionally graded additive manufacturing. The objective of this document is to present a conceptual understanding of Functionally Graded Additive Manufacturing (FGAM).
- ISO/ASTM 52911-1:2019 Additive manufacturing—Design—Part 1: Laser-based powder bed fusion of metals. This standard defines the characteristics of the laser-based powder bed fusion of metals (PBF-LB/M) and contains detailed design recommendations.
- ISO/ASTM 52911-2:2019 Additive manufacturing—Design—Part 2: Laser-based powder bed fusion of polymers. This document defines the characteristics of the laser-based powder bed fusion of polymers (LB-PBF/P) and contains detailed design recommendations.
- ISO/ASTM 52902:2019 Additive Manufacturing—Test Artifacts—Geometric capability assessment of additive manufacturing systems. This standard contains a general description of model specimen geometries, together with the quantitative and qualitative computing to be conducted on a test specimen(s) in order to assess the efficiency of additive manufacturing (AM) systems.

Additionally, the Committee for Standardization continually works on further standards applicable to additive manufacturing.

## 3. Research Methodology

### 3.1. Division of Models According to Their Intended Use

On the basis of work conducted in the Rapid Prototyping System Laboratory of the Rzeszów University of Technology, the authors divided additive manufacturing products according to their intended use:

(1) Models that are manufactured for the purpose of visual presentation, including the following:
  - Conceptual prototypes: models that present the simplified constructional and functional assumptions of a product; any technique can be applied to manufacture them in order to present a general conception relatively rapidly and economically. The basis for making it may be a product sketch made by, for instance, a fine artist.
  - Numerical prototypes: models that are developed in the software environment. Numerical prototypes include a model or a set of models to be visualized for kinematic simulations, load simulations, preparation of data for manufacturing, and verification based on computer-assisted systems.
  - Visual prototypes: models that present the actual dimensions or an assumed scale, geometric characteristics, and the colors and/or quality of a product surface.
  - Ergonomic prototypes: models that specify the conditions of a product functioning in its intended environment in reference to its future users, taking into account ergonomic technical assumptions.
(2) Models that are directly connected to the manufacturing process, which are divided into the following categories:
  - Technological prototypes: models that are used to develop and verify technological assumptions for the manufacturing process of a product. Technological prototypes may be developed for and specific to particular stages of the technological process.
  - Construction prototypes: models that are intended for a comprehensive assessment of a construction solution on the basis of target functionality and the assumptions of a highly detailed technological process.
  - Functional prototypes: models that enable the assessment of the main product functions in conditions similar to the actual ones, taking into account operating processes in a simplified configuration. They may be made from materials with properties that are similar to those of materials of the end product.
  - Technical prototypes: models that have any or all characteristics (functional and visual) of an end product, so they can be tested in real operating conditions. These models are fabricated with the materials used to make the end product. This makes it possible to prepare the target technological process for manufacturing conditions.

### 3.2. Developing Algorithms of Quality Control Processes for Particular Model Groups

(1) Algorithm of the quality control process for models manufactured for the purpose of visual presentation

**Figure 1:**
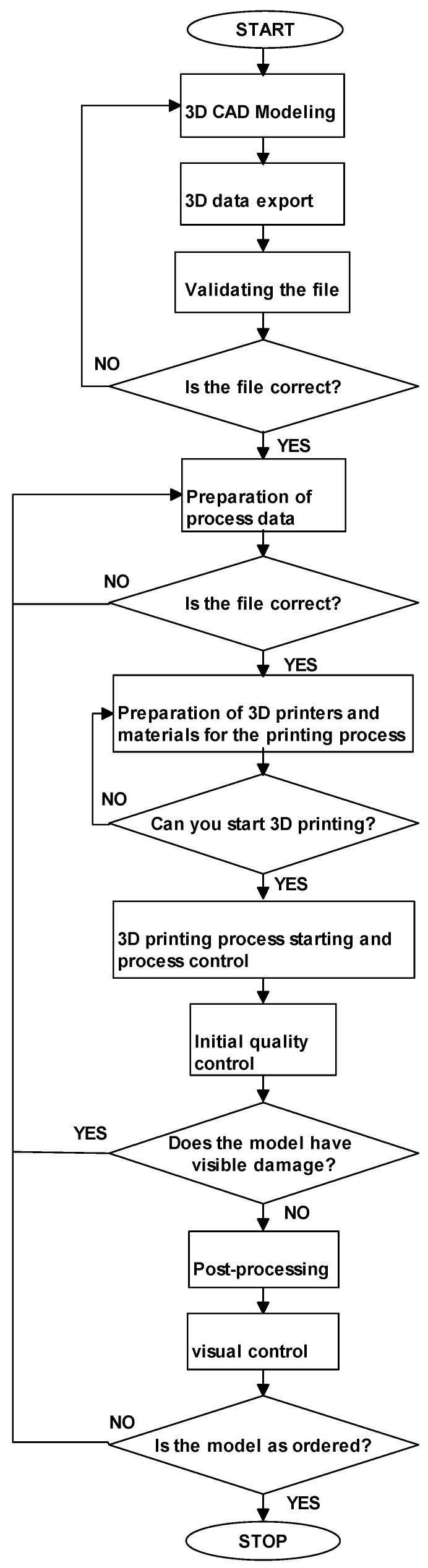
Algorithm of the quality control process for models manufactured for the purpose of visual presentation.

A good practice of enterprises in the 3D industry is to develop an algorithm for completing a work order for a 3D production model, as well as activities related to quality control. The algorithm presented in Figure 1 may be a sui generis mode of action for manufacturing models intended for visual presentation, in which dimensional and shape accuracy is less significant.

In the algorithm presented in Figure 1, the manufactured 3D model is checked to ensure the correctness of the designed 3D CAD models and confirm the prepared process data, and only visual control is conducted.

(2) Algorithm of the quality control process for models that are directly connected to the manufacturing process

**Figure 2:**
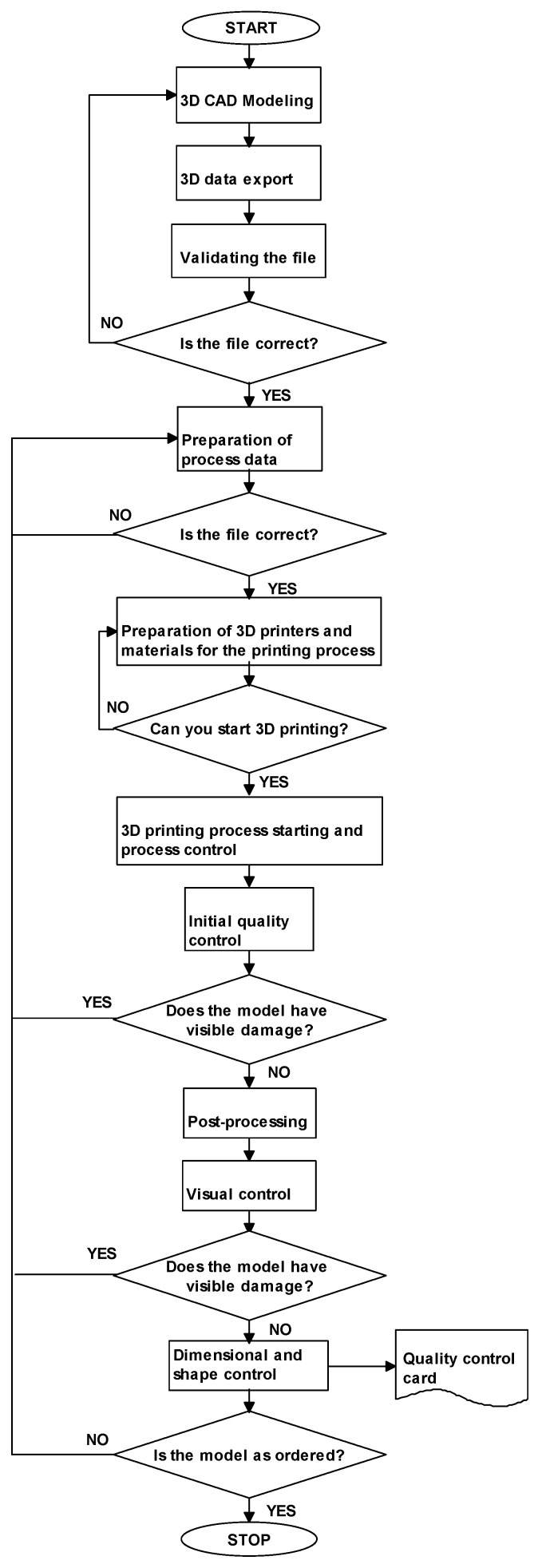
Algorithm of the quality control process for models that are directly connected to the manufacturing process.

The algorithm presented in Figure 2 is specific to models that are directly connected to the manufacturing process. In this case, it is very important to verify the dimensional and shape accuracy of the product.

For manufacturing models that are directly connected to the manufacturing process, visual control is the first stage of verifying quality compliance after manufacturing a physical artifact, and the result determines further courses of action. If damage to the model is visible to the ‘naked eye’, then it is usually treated as a non-compliant product. Then, the discovered errors are analyzed, and the printing process has to be recommenced. If no such damage is apparent, then additional quality verification activities are conducted by means of measuring instruments, gauges, automated and manual scanners, tomography, or coordinate computing techniques. Therefore, the course of 3D print quality control may be highly tailored and differ between products.

### 3.3. Proposal of the Quality Control System

**Figure 3:**
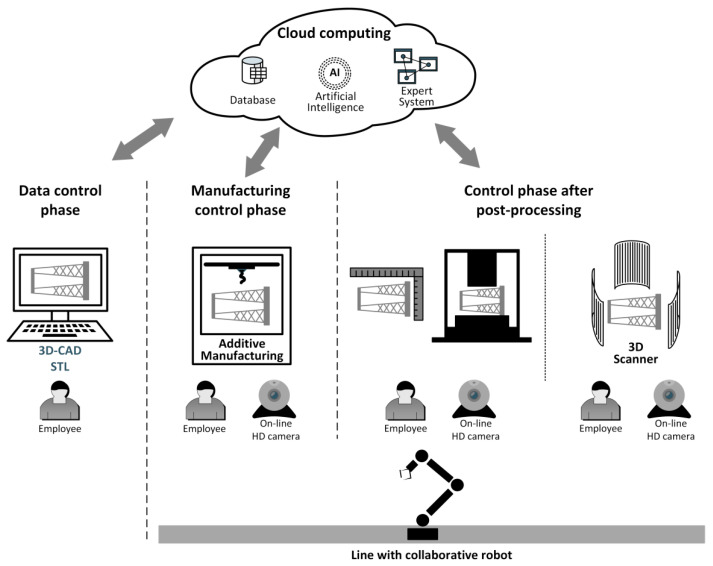
Manufacturing process control in additive manufacturing in the context of Industry 4.0.

In the course of this work, the authors developed a system that can be applied to various quality control methods and is adaptable to different measuring instruments and manufacturing process supervision methods. The general concept of the proposed system is presented in Figure 3. Its key feature is its readiness to be completely integrated with ICT solutions within the framework of Industry 4.0. This system is divided into three separate control phases: data control (3D CAD models and process data), visual manufacturing control, and post-processing control. Post-processing control is further divided into two stages: control with specialized measuring instruments and control with a contactless optical system.

The proposed architecture of the comprehensive system for additive manufacturing control accounts for both direct control by qualified specialists and complete automation, as well as remote supervision, to meet the needs of Industry 4.0. Both forms of supervision can be applied simultaneously: one to meet in-house needs and the other in the case of supervision by a customer. In particular, remote supervision has enormous potential within the scope of dispersed production lines, which are typical in the context of the global economy [32,38,39]. Furthermore, the presented system architecture involves local repositories of product and component quality records, and it collects, synchronizes, and processes data in a computing cloud. Thus, the features of the proposed system meet the needs of Industry 4.0, as it combines and integrates individual design and manufacturing processes, available knowledge, and the experience and skills of a dispersed team of specialists. The fulfillment of these needs is aimed at supporting diverse production systems and increasing the availability and efficiency of these processes with the use of currently available computer network technologies, IT systems, and the automation and control of manufacturing processes. Therefore, this system meets the requirements of Industry 4.0 in all indicated areas. It enables the integration of remote resources of both individual elements and entire lines, the use of information compiled in knowledge bases, and the use of modern systems based on artificial intelligence. Moreover, it is adapted to exploit the potential for remote operation and consultation. Additionally, the proposed architecture has features of open systems, including scalability, customization, and interoperability between different systems and resources.

This system was implemented in the Rapid Prototyping System Laboratory of the Rzeszów University of Technology under conditions of complete automation and remote supervision. The proposed IT system enables remote access to design tools and software for supervising the operation of a 3D printer. Within this system, the storage of digital quality records in a cloud was developed in order to ensure constant access to, and verification of, data in all phases of process control. Therefore, possible deviations from the initially set values of quality parameters can be rapidly and remotely assessed. The manufacturing and post-processing phases were integrated with the application of a robot (Universal Robot UR3, Universal Robots, Katowice, Poland).

The described approach ensures the 24/7 automation of the control process and, simultaneously, enables the completion of work despite restrictions connected to the SARS-CoV-2 pandemic. Additionally, applying a rotating high-resolution camera ensures the maintenance of constant visual control [40,41]. The contemporaneous use of cameras can ensure a high approximation factor, and thus, quality control can be performed in the course of printing and in the post-processing phase. To this end, a turntable, among other equipment, is used during operation to enable the accurate (visual) verification of a manufactured component from every direction. Due to the large approximation factor, inaccuracies that are invisible to the human eye can be captured with the application of specialized tools prior to subsequent stages of control. Early identification of such deviations can reduce the cost and time involved in further control processes.

**Figure 4:**
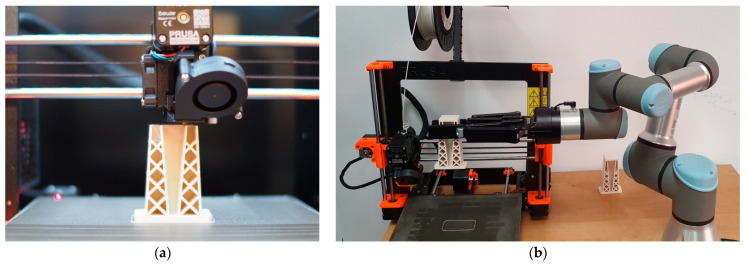
(**a**) Process control with the application of a camera; (**b**) Collaborative robot (Universal Robot UR3) with a 3D printer.

Figure 4a depicts a control process with the application of a camera. In Figure 4b, a collaborative robot working with a 3D printer (Prusa i3 MK3S) is presented.

In this study, the presented system was applied to the manufacturing of a car mirror.

As presented in Figure 3, the architecture of the control system can include specialized IT tools such as export systems and AI mechanisms, which, in the future, will support control processes, particularly those in the specialized production of elements for the aviation and automotive industry.

## 4. Quality Control of a Car Mirror Holder

**Figure 5:**
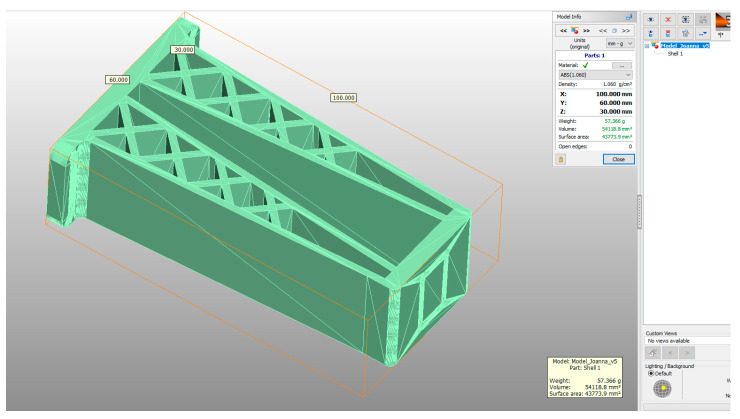
The 3D model (CAD) of a car mirror holder in the 3D-Tool program.

A 3D CAD model of a car mirror holder was applied in the analysis of print quality control. The authors’ intention was to ensure that the chosen element was universal enough to be used by companies in the automotive industry. When commencing the rapid prototyping process, the dimensions of the designed artifact should be controlled (verify if the model was not scaled). Similarly, it is very important to conduct an initial verification of the model after data processing; in this study, initial verification was performed with the 3D-Tool program (Figure 5).

The subsequent stage was the selection of methods and materials for printing. The materials used for printing were the RGD720 photopolymer resin (applied method: PolyJet), ABS M30 thermoplastic (applied method: FDM), E-Partial photopolymer resin (applied method: DLP), PLA thermoplastic (applied method: FFF), and ABS thermoplastic (applied method: MEM). The materials used for printing, together with the model weight, thickness, and area, are presented in Table 1.

In the manufacturing process, the following 3D printers were applied: Object Eden 260V, 3D STRATASYS F170, EnvisionTEC Vida, Prusa i3 MK3, and UP BOX+.

The prototype was included in the group of models that are directly connected to the manufacturing process. Then, the quality control process for models after post-processing was divided into three stages: visual control, control with a caliper, and control with a contactless optical system. The computing techniques were selected on the basis of the specific profile and the intended use of the model, as well as financial aspects.

The measuring tools used to control the dimensional and shape accuracy should be selected in such a way as to ensure that reliable results are obtained. It is necessary to maintain an appropriate level of accuracy in the measurement process, which will eliminate possible measurement errors while minimizing uncertainty. For these reasons, the applied measuring devices had an accuracy of at least an order of magnitude higher than that of the manufacturing technologies used. Therefore, the analysis of measurement errors was unnecessary, and the dimensional and shape accuracy of the research models could be reliably assessed.

### 4.1. Visual Control

Visual control constitutes the most cost-effective qualitative assessment method. It does not require costly equipment, nor does it result in the destruction of the assessed item. Despite its numerous assets, visual control is not flawless, principally because it is impossible to present the obtained results in SI units. It should also be noted that visual control entails some bias, as it depends on a given employee’s predispositions and expertise; therefore, correctness is not guaranteed with this method [42].

Visual control is the most widely used nondestructive technique because it is simple and can be conducted quickly [13]. In principle, visual investigation is the first to be conducted among all qualitative assessments. Thus, the research models were verified by checking for imperfections that were visible to the naked eye. In this research project, the visual investigation was conducted to assess the following model characteristics: general model representation, surface deformations, surface state, ‘cobweb effect’, layer relocation, and broken sections.

In Table 2, photographs from the visual control process are presented. The initial visual assessment of the five 3D models did not reveal any imperfections or damage that would have made them unsuitable.

### 4.2. Control with the Application of a Caliper

The next stage of the quality control study was the dimensional control of the models, which was conducted with an electronic caliper, the accuracy of which was 0.02 mm. In order to determine the dimensional compliance of the models, four typical dimensions were measured and compared with tolerance parameters. The results are presented in Table 3.

### 4.3. Control with the Application of a Contactless Optical System

The accuracy of the geometric shape of the manufactured research models was analyzed with a contactless optical system based on a coordinate optical scanner (ATOS Triple Scan II Blue Light of the GOM company). For the data analysis, the software of an ATOS Professional V7 scanner was applied. By applying blue light, this scanner enables virtual computing regardless of the intensity of daylight or artificial (white) light. Moreover, it can significantly reduce the computing time owing to the turntable, among other components, integrated with the ATOS computing system. Automation significantly accelerates the computing process because it partially eliminates the need to manually reposition the scanned artifact. The methodology of contactless measurements with the use of the Atos II Triple Scan 3D optical scanner was developed after many trials to determine the appropriate measurement process. A strategy was adopted in which two independent measurement series were carried out in two positions of the target in relation to the measuring table. The process was configured so that each of the two series was measured with the part placed in a given plane of the table and with the plane rotated by 180 degrees. However, this required reference points to be distributed so that at least three were visible in both measurement series. This enabled the program to compile measurement data obtained in both measurement series, thereby obtaining information about the entire measured geometry. This significantly reduced the duration of the digitization process, and based on the inspection of the geometry of the scanned model, the appropriate number of steps for dividing the full rotation of the measuring table for a single measurement series was determined.

By applying the GOM Inspect V8 program, 3D maps of the deviations of actual model surfaces from the designed model were plotted, and two files were output: the first was in STL format, which was exported from CAD software, and the other was a 3D grid reflecting the actual geometry of the research models and representing the models measured by an optical 3D scanner. The developed 3D CAD models are also applicable to quality control processes based on coordinate optical scanners, where an industrial robot can be used. This approach eliminates the need to manually adjust the specimen during the measurement process, which significantly shortens the inspection stage. Due to such a measurement system not being available in this study, this solution was omitted. Nevertheless, the presented measurement methodology allows for the implementation of such a control procedure.

On the basis of the presented issues and a number of previous studies, optical measurements were used in this study. Data visualization and the capabilities of the GOM Inspect software provide advantages over the use of contact measurements. Having the complete geometry of the research models obtained during the measurement process, rather than only cross-sections or a point cloud obtained by contact methods, makes it much easier to apply corrections. Due to the effective integration of CAD/CMM/RP systems, a more effective process for minimizing geometric errors can be implemented using data obtained from optical scanning.

In order to determine the accuracy of the actual geometry, analyses presented in the form of color deviation maps were conducted. The results of the analyses are presented in Table 4. The conducted analyses were focused on the geometry of the research models and were performed in order to assess the dimensional and shape accuracy. Global geometry analyses were performed. On the basis of the results, deviation values were obtained at the selected inspection points.

The presented results of the geometric analysis of car mirror holder models show imperfections that were not identified at the earlier stages of quality control.

The research results indicate that the most precise method is PolyJet. However, this technology is the most expensive of those described in this paper; therefore, every time a 3D printing method is selected, its cost-effectiveness should be evaluated. The data reported above also reveal that FFF has the optimal price–quality ratio. Due to its price and straightforward operation, the Prusa i3 MK3 printer is popular with both regular consumers and legal entities. MEM is the least precise representation, as an uneven surface and significant deviations are clearly visible.

## 5. Conclusions

The quality control methodology presented in this article, developed on the basis of a car mirror prototype, can be successfully applied to products manufactured additively from polymeric materials. The tests and analyses reveal that the intended use of the prototype is important, and verifications should be carried out in the following order:

- verification of the correctness of the numerical data of the 3D CAD model,
- confirmation of process data intended directly for 3D printing (analysis of support structures, layers, and transition paths in subsequent layers),
- verification of the correctness of model construction during incremental process, carried out directly or remotely,
- visual inspection of the prototype immediately after its construction,
- visual inspection after the completion of post-processing,
- dimensional and shape accuracy control with the use of measuring tools,
- dimensional and shape accuracy control with the use of computer-controlled measuring machines,
- confirmation of the accuracy and internal structure with the use of computer tomography.

The application of 3D scanners to quality control makes it possible to complete the manufacturing process using numerical data from computing. As a result, the geometric accuracy of a product can be completely documented, records can be created, and remedial actions can be prepared. This approach is compatible with manufacturing systems in the context of Industry 4.0. Additionally, certain computing systems, such as those of the GOM company, come with special-purpose applications that allow a 3D STL model to be corrected using data from the computing process, resulting in a product with improved dimensional and shape accuracy.

In summary, models that are fabricated with additive manufacturing may be subjected to different quality control processes depending on their intended use, which should thus be considered when selecting quality assessment methods. It is also important to ensure that cost aspects are always taken into account, as not all products require high precision and durability.

## Figures and Tables

**Table 1:** Materials applied in the printing of particular models, presented as designed in the 3D-TOOL program.

RM1	RM2	RM3	RM4	RM5
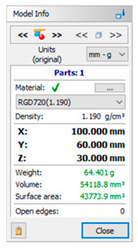	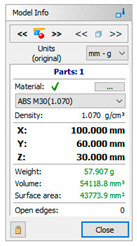	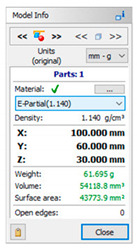	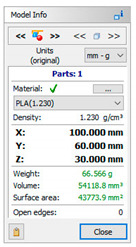	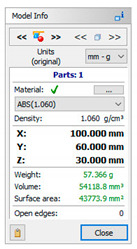

**Table 2:** Photographs from visual control.

No.	Description	Symbol	View
1.	A car mirror holder made with the application of the PolyJet method; material: RGD 720	RM1	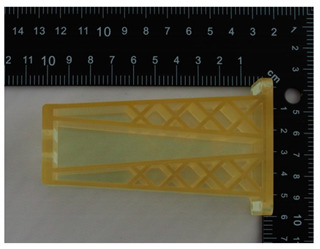
2.	A car mirror holder made with the application of FDM; material: ABS M30	RM2	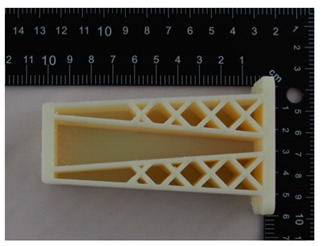
3.	A car mirror holder made with the application of the DLP method; material E-Partial	RM3	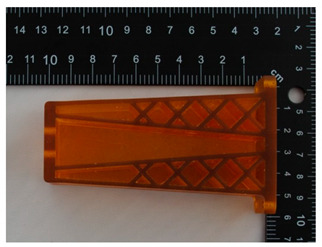
4.	A car mirror holder made with the application of FFF; material: PLA	RM4	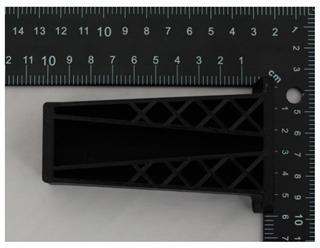
5.	A car mirror holder made with the application of MEM; material: ABS	RM5	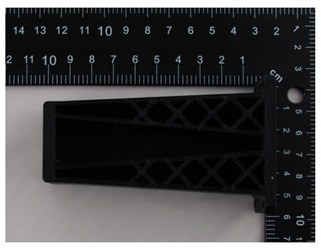

**Table 3:** Results of model dimension control.

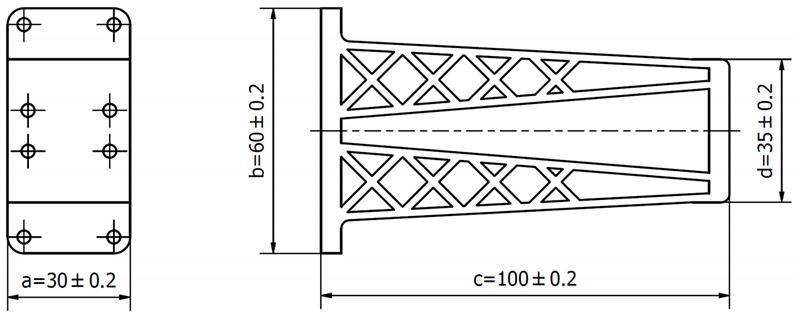							
**Model**	**Name**	**Measured** **Value**	**Unit**	**Tolerance**	**Actual Value (** **Mean of 3 Measurements)**	**Deviation**	**Compliance**
RM1	Dimension a	30.00	mm	0.20	29.89	−0.11	OK
RM1	Dimension b	60.00	mm	0.20	60.00	0.00	OK
RM1	Dimension c	100.00	mm	0.20	100.13	0.13	OK
RM1	Dimension d	35.00	mm	0.20	35.03	0.03	OK
RM2	Dimension a	30.00	mm	0.20	30.28	0.28 ↑	NOK
RM2	Dimension b	60.00	mm	0.20	60.13	0.13	OK
RM2	Dimension c	100.00	mm	0.20	100,18	0.18	OK
RM2	Dimension d	35.00	mm	0.20	35.12	0.2	OK
RM3	Dimension a	30.00	mm	0.20	29.85	−0.15	OK
RM3	Dimension b	60.00	mm	0.20	59.78	−0.22 ↓	NOK
RM3	Dimension c	100.00	mm	0.20	99.73	−0.27 ↓	NOK
RM3	Dimension d	35.00	mm	0.20	34.90	−0.10	OK
RM4	Dimension a	30.00	mm	0.20	29.96	−0.04	OK
RM4	Dimension b	60.00	mm	0.20	59.80	−0.20	OK
RM4	Dimension c	100.00	mm	0.20	99.76	−0.24 ↓	NOK
RM4	Dimension d	35.00	mm	0.20	34.93	−0.07	OK
RM5	Dimension a	30.00	mm	0.20	30.15	0.15	OK
RM5	Dimension b	60.00	mm	0.20	60.04	0.04	OK
RM5	Dimension c	100.00	mm	0.20	99.72	−0.28 ↓	NOK
RM5	Dimension d	35.00	mm	0.20	34.83	−0.17	OK

Notes: sign ↑ means that the measured value is above the tolerance range, sign ↓ means that the measured value is below the tolerance range.

**Table 4:** Maps of deviations in the accuracy of the research models.

**Isometric View RM1**
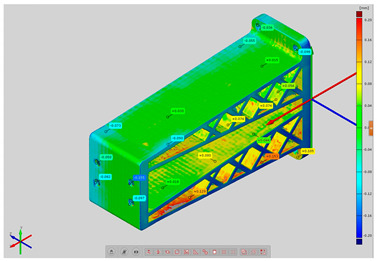
**Isometric View RM2**
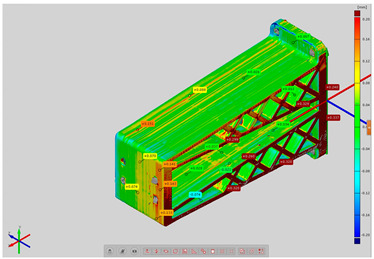
**Isometric View RM3**
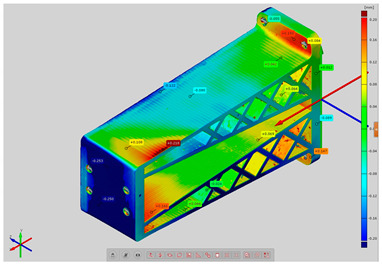
**Isometric View RM4**
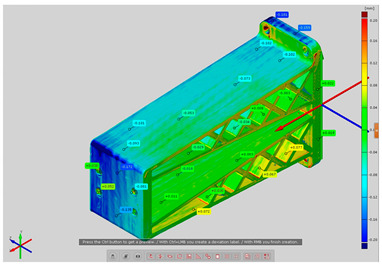
**Isometric View RM5**
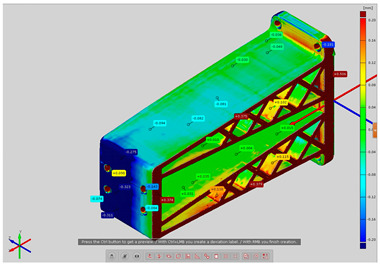

## Data Availability

Not applicable.
